# Increased iron bioavailability from lactic-fermented vegetables is likely an effect of promoting the formation of ferric iron (Fe^3+^)

**DOI:** 10.1007/s00394-015-0857-6

**Published:** 2015-02-12

**Authors:** Nathalie Scheers, Lena Rossander-Hulthen, Inga Torsdottir, Ann-Sofie Sandberg

**Affiliations:** 1 Department of Biology and Biological Engineering, Food and Nutrition Science, Chalmers University of Technology, 412 96 Gothenburg, Sweden; 2 Department of Clinical Nutrition, Sahlgrenska Academy, University of Gothenburg, Box 459, 405 30 Gothenburg, Sweden; 3 Faculty of Food Science and Nutrition, University of Iceland and Unit for Nutrition Research, National University Hospital, Reykjavík, Iceland

**Keywords:** Lactic fermentation, Iron, Absorption, Ferric, Bioavailability

## Abstract

**Background:**

Lactic fermentation of foods increases the *availability* of iron as shown in a number of studies throughout the years. Several explanations have been provided such as decreased content of inhibitory phytate, increased solubility of iron, and increased content of lactic acid in the fermented product. However, to our knowledge, there are no data to support that the *bioavailability* of iron is affected by lactic fermentation.

**Objectives:**

The objective of the present study was to investigate whether the bioavailability of iron from a vegetable mix was affected by lactic fermentation and to propose a mechanism for such an event, by conducting human and cell (Caco-2, HepG2) studies and iron speciation measurements (voltammetry). We also investigated whether the absorption of zinc was affected by the lactic fermentation.

**Results:**

In human subjects, we observed that lactic-fermented vegetables served with both a high-phytate and low-phytate meal increased the absorption of iron, but not zinc. In vitro digested fermented vegetables were able to provoke a greater hepcidin response per ng Fe than fresh vegetables, indicating that Fe in the fermented mixes was more bioavailable, independent on the soluble Fe content. We measured that hydrated Fe^3+^ species were increased after the lactic fermentation, while there was no significant change in hydrated Fe^2+^. Furthermore, lactate addition to Caco-2 cells did not affect ferritin formation in response to Fe nor did lactate affect the hepcidin response in the Caco-2/HepG2 cell system.

**Conclusions:**

The mechanism for the increased bioavailability of iron from lactic-fermented vegetables is likely an effect of the increase in ferric iron (Fe^3+^) species caused by the lactic fermentation. No effect on zinc bioavailability was observed.

## Introduction

Lactic fermentation is one of the oldest methods for preserving foods. It is becoming increasingly popular since the fermentation increases the nutritional value of foods and that consumers perceive it as natural and free of food additives. Commercial starter cultures are available for everyone to perform well-controlled fermentations at home. During lactic fermentation, lactic acid and other organic acids are produced, which lowers the pH and gives rise to the sour taste. A number of health effects have been associated with lactic-fermented foods such as improved control of intestinal infections [1], improved digestion of lactose [2], and control of serum cholesterol levels [1]. We have previously found that a mix of fresh or fermented vegetables (carrots, turnips, and cabbage) attenuated postprandial glycaemia after a composite meal including high-glycaemic foods and that the fermented vegetables had a greater attenuation effect [3].

Lactic fermentation of maize, soybeans, and sorghum reduces the content of phytate, a well-known inhibitor of iron and zinc absorption [4]. Organic acids, in particular lactic acid, have been proposed to be an inhibitor [5, 6] as well as an enhancer of iron absorption [7]. Sauerkraut, a lactic-fermented cabbage, has been observed to enhance iron absorption from a meal [8]. Previous studies done in our laboratory indicated that lactic fermentation of white sorghum and maize gruels yields an almost complete degradation of phytate which improves iron solubility [9]. In addition, we have observed that in vitro digested fermented carrot juice was associated with increased solubility of iron compared to fresh juice [10]. There are obviously several indicators on that lactic fermentation should be beneficial for the absorption of iron and possibly other minerals. However, the responsible mechanisms are unclear. It is obvious that increased soluble iron means more iron available for absorption, but not if it means that iron *bioavailability* is increased by the lactic fermentation.

In the present study, we are reporting our work on the absorption of iron from lactic-fermented vegetables. We started by investigating the effects of lactic-fermented vegetables on the iron and zinc absorption of a low- versus high-phytate meal in humans. We compared the content of ferric (Fe^3+^) and ferrous (Fe^2+^) iron in the in vitro digested vegetable mixes to estimate whether the iron speciation differed between the fermented and fresh vegetables. Further studies were made in the Caco-2/HepG2 cell system to investigate whether hepatocyte hepcidin release was affected by the lactic fermentation. To address the effect of lactic acid as a major product from lactic fermentation, we also tested whether ferritin formation and hepcidin production in Caco-2 and Caco-2/HepG2 models, respectively, were influenced by the presence of lactic acid.

## Materials and methods

### Human iron absorption from lactic-fermented vegetables

#### Subjects

In total, 17 healthy volunteers, 11 men and 6 women aged 21–54 years, participated in the study. In each group, there were both men and women and three of the subjects were regular blood donors. All subjects were informed both orally and in written words about the aims and procedures of the study. The study was approved by the Radioisotope Committee and the Ethical Committee of the Sahlgrenska University Hospital.

#### Lactic fermentation

A mix of raw vegetables consisting of carrots (40 %), turnips (25 %), white cabbage (15 %), and equal parts of parsnip, celery, and onion (20 % in total) was used in the study. A starter culture of *Lactobacillus plantarum* [0.05 g (2.4 × 10^9 ^CFU); Vegestart-10, CHR, Denmark] in sodium chloride solution (1.5 %; 200 mL) was added to the grated vegetables (500 g) in fermentation pots. The lactic acid bacteria were allowed to ferment for 1 week at 20 °C. The fermented vegetables were then stored for 3 weeks at 4 °C before use.

#### Test meals

Two different test meals to go with the vegetables were designed to contain either high or low amounts of phytate. The first meal consisted of two bread rolls made of wheat flour (55 % extraction rate), which was ingested with 100 g of fermented or fresh vegetables. The second meal was bread rolls made of wheat bran (6.3 %) and wheat flour (93.7 %). Each roll was made of 40 g of flour and was leavened for 50 min. The dough was extrinsically labelled with ^55^Fe (37 kBq) and ^59^Fe (37 kBq) per roll. Test meals (a) and control meals (b) were served on alternate mornings after an overnight fast on four consecutive days in the order ABBA or BAAB. A blood sample was drawn 2 weeks after serving the last meal.

#### Oral reference dose

A solution of hydrochloric acid (0.01 M, 10 mL) containing Fe as FeSO_4_ (3 mg) and ascorbic acid (30 mg) labelled with ^59^Fe was used as a reference in the study. The vials (10 mL) containing the Fe solution were rinsed twice with water, which was also consumed by the subjects. Each subject received two reference doses (total dose of 55.5 kBq ^59^Fe) on two consecutive mornings after overnight fast to eliminate day-to-day variations. No food or drink was allowed for 3 h after the reference dose. The reference doses were given 14 days after the test meal, and the absorption of ^55^Fe and ^59^Fe was measured 2 weeks later (28 days in total) in blood samples (^55^Fe and ^59^Fe) and by whole-body counting (^59^Fe).

#### Iron absorption measurements

The relative absorption of iron (the bioavailability) was estimated by calculating the absorption of ^55^Fe from the meals, by comparing the ratio of ^55^Fe (from meals) and ^59^Fe (from reference dose) in blood samples to the whole-body count of ^59^Fe. Analysis of ^55^Fe and ^59^Fe in blood was made according to Eakins and Brown [11] using a liquid scintillation spectrometer (Tri-carb, Packard instruments, San Antonio, Texas, USA). All procedures and calculations were described previously [12].

### Human zinc absorption from lactic-fermented vegetables

#### Subjects

Fourteen healthy subjects, 11 men and 3 women aged 24–55 years, participated in the study. Their serum zinc levels were within normal range (14.0–20.2 μM). Informed subject consent was obtained, and the study was approved by the Radioisotope Committee and the Ethical Committee at Sahlgrenska University Hospital.

#### Test meals

As in the iron absorption study, two different test meals consisting of two bread rolls each made of either wheat flour (60 % extraction rate) or wholemeal wheat flour were investigated. The dough was labelled with ^65^Zn at 0.05 MBq per 30 g of used flour (Amersham International, Amersham, Buckinghamshire, UK). Each subject was served two of these rolls together with cottage cheese (50 g) and fresh or fermented vegetables (200 g). Cottage cheese was included in this study to mimic a composite meal.

#### Zinc absorption measurements

Zinc absorption was determined using the radionuclide technique described by Arvidsson et al. [13]. The activity of each wheat roll was measured in the whole-body counter before serving. Each subject’s background radiation was also measured in the whole-body counter before intake of the labelled test meal. The meals were served as breakfast. No food or drink was allowed during the 12-h period before breakfast or 3 h after the intake of the test meal. The whole-body retention was measured once, 10–14 days after intake to allow excretion of the unabsorbed fraction of the isotope. The time for the excretion of the initially absorbed isotope was estimated according to Arvidsson et al. [13] who measured the excretion rate of an intravenously administered dose of ^65^Zn in a similar group of subjects. Each subject participated twice serving as his or her own control, and the order of serving either lactic-fermented or fresh vegetables was randomized. During the first 10 days, 11 ± 0.3 % of the absorbed dose was excreted, and the mean excretion from day 10 to day 30 was 10 %.

### Analysis of mineral, phytate, and organic acid content in the test meals

Freeze-dried bread rolls, fresh and lactic-fermented vegetables were analysed in duplicates for their contents of Zn, Fe, and Ca. Glassware was washed in acid and rinsed in deionized water before being used. Zn and Fe content was determined by atomic absorption spectrophotometry (AAS; Perkin-Elmer Model 360, Norwalk, CT) after dry ashing at 450 °C. Ca and Mg were determined by AAS after wet ashing (290–300 °C, 15 min.) in concentrated H_2_SO_4_ and hydrogen peroxide after the addition of lanthanum oxide (5 %). The same digest was used to determine P according to Harland and Oberleas [14]. Reference standards for Zn, Fe, Ca, and Mg were prepared from Titrisol (Merck, Darmstadt, Germany). Reference standard material [Bovine Liver SRM 1577(a), National Bureau of standards, Gaithersburg, MD] for Zn and Fe was run simultaneously and fell within the certified range (Zn: 124.1 ± 3.9 mg/kg, Fe: 177.6 ± 7.8 mg/kg). Reference material from our laboratory was used for the control of the Ca and Mg analysis. The reference material was made from large batches of freeze-dried foods, which were compared to known standards and used for all routine analyses of Ca and Mg. The variation coefficients for these materials were 4.1 (*n* = 11) for Ca and 3.7 (*n* = 16) for Mg.

Phytate and its degradation products were determined according to Sandberg et al. [15, 16]. Briefly, the samples were extracted with HCl (0.5 M) for 3 h. The inositol phosphates were separated from the crude extract by ion exchange chromatography and determined by ion pair C18 reversed phase HPLC using formic acid/methanol and tetrabutylammonium hydroxide in the mobile phase. All analyses were made at least in duplicates. The analysis was performed using a HPLC pump (Waters Model 510, Milford, MA, USA) equipped with a C18 Kromasil (5 μm) column (15 cm, 2 mm i.d.). The inositol phosphates were detected and quantified by refractive index (ERC-7510 RI-Detector, Erma Optical Works Ltd., Japan). Retention times and peak areas were measured using a Hewlett Packard analytical data system (HP 3350, Agilent Technologies, Talo Alto, CA, USA). Injections were made with a Poole 20-μl loop.

Organic acids in fresh and fermented vegetables were determined according to Ashoor and Knox [17] with modifications. Vegetables (50 g) and deionized water (50 mL) were homogenized (Braun MR 400 HC, Kronberg, Germany) and left to equilibrate for 1 h. The mix was centrifuged (3000 rpm; 5 min), and the supernatant was filtrated through 45-μm filters (GHP Acrodisc, Gelman Sciences, MI, USA). A sample of 60 μl was injected and quantitatively determined by HPLC. The HPLC ion exclusion chromatography system consisted of a Waters 510 pump (Milford, MA, USA), a CMA 200 microsampler (Stockholm, Sweden), an Aminex column HPX-87H (BioRad Lab., Richmond, CA, USA), and UV absorption detection at 210 nm (HP 1050, Agilent Technologies, Palo Alto, CA, USA). The conditions for the HPLC analysis were as follows: mobile phase: 0.008 M H_2_SO_4_, flow rate: 0.6 mL/min, column temperature: 65 °C. Borwin chromatography software (JMBS Developpments, Le Fontanil, France) was used for the processing of peaks. Citric acid, l(−)-malic acid, l-lactic acid sodium salt, acetic acid, and propionic acid (all from Fluka, Sigma-Aldrich, Stockholm, Sweden), and succinic acid (Merck, Darmstadt, Germany) were used as external standards. Ascorbic acid in raw and lactic-fermented vegetables was determined by titration with 2.6-dichloroindophenol according to AOAC.

### Lactate and fermented vegetable experiments in the Caco-2/HepG2 cell system

#### Sample preparation

The fresh and fermented vegetables were prepared in the same way as in the human iron and zinc absorption studies except for that another starter was used containing a mix of *L. plantarum, L. mesenteroides,* and *L. pentosus* (Body Ecology Veggie starter). The vegetable mixes were in vitro digested and filtered according to standard procedure as described in [18].

#### Cell lines and set-up of the cell model

Human Caco-2 cells (HTB37) and human HepG2 cells were obtained from American Type Culture Collection (Rockville, MD, USA) in passage 19 and 74, respectively. Stock cultures of Caco-2 cells were maintained in Dulbecco’s modified α essential medium (DMEM) and HepG2 cells in minimal essential medium (MEM) with Earle’s salt, and both media were supplemented with FBS (16 %). At passage 35–38, the Caco-2 cells were seeded in 12-well plates on Transwell^®^ polycarbonate inserts (0.4 µm) at 60,000 cells/insert. Simultaneously, the HepG2 cells were seeded at the bottom of 12-well plates at passage 80–84 at 200,000 cells/well. The antibacterial, antifungal, and antimycoplasma agent Normocin™ (Invivogen, CA, USA) was added to the medium after plating. Thirteen days after seeding, the inserts with Caco-2 cells were combined with the wells containing the HepG2 cells in MEM (FBS 1 %). The combined cell lines were allowed to equilibrate for another 24 h before the experiments.

#### Cell experiments including hepcidin, ferritin, and total protein analysis

Caco-2/HepG2 cells in triplicates were treated with 1) Fe (40 μM) and lactic acid at 0, 0.2, 0.8, and 1.6 mg/well for 2 h and 2) with filtered digested fermented or filtered digested fresh vegetables (250 µl) for 3 h. The filtration was done to simulate the filtering effect of the mucus layer. The medium was aspirated and replaced by fresh MEM (FBS 1 %) and left in the incubator for another 22 and 21 h, respectively, to allow for ferritin formation in the Caco-2 cells and hepcidin production in the HepG2 cells. Then, the basal medium was collected for hepcidin analysis. The Caco-2 cells were lysed in RIPA buffer (Sigma-Aldrich, Schnelldorf, Germany) for ferritin and total protein estimation. Hepcidin and ferritin content was measured by commercial antibody-based kits according to the manufacturers’ instructions (Bachem, Torrance, CA, USA and DRG, GmbH, Germany, respectively).

### Lactate experiments in mono-cultured Caco-2 cells

#### Cell line

Caco-2 cell culture (HTB-37) was obtained from the American Type Culture Collection (Rockville, MD) at passage 19. Stock cultures were maintained in DMEM supplemented with FBS (16 %), non-essential amino acids (1 %), penicillin and streptomycin 100 U/L and 10 mg/mL, respectively. The cells were seeded in 12-well transwell^®^ plates (Corning, Tewksbury, MA, USA) at 60,000 cells/insert. All experiments were performed 14 days post-seeding.

#### Lactate and iron uptake experiments

Eleven days post-seeding, the medium was changed to MEM containing penicillin and streptomycin 100 U/L and 10 mg/mL, respectively. No other supplements were present in the medium. The apical or basal medium was pre-incubated with lactate (500 μM final concentration) for 4 h followed by the apical addition of Fe (20 μM). Controls with only MEM (negative control), lactate, and Fe were also included. After 2 h of Fe incubation, the medium was aspirated and new unsupplemented MEM was added. The cells were brought back to the incubator for another 22 h before harvest. Cells were lysed in cold RIPA buffer (Sigma-Aldrich, Sweden) containing EDTA-free protease inhibitor cocktail at 40 μL/mL of lysis buffer (Roche, Switzerland). Ferritin analysis was performed by a Coat-A-Count Ferritin IRMA kit (DPC, Denmark) and counted for 20 min in a CG400 γ-counter (Intertechnique, France). Total cellular protein content was estimated by the bicinchoninic acid (BCA) assay (Pierce, Sweden).

### Analysis of Fe^3+^ and Fe^2+^ using differential pulse anodic stripping voltammetry (DPASV)

The iron speciation (Fe^3+^ and Fe^2+^) of in vitro digested fermented and fresh vegetables was analysed with differential pulse anodic stripping voltammetry (DP-ASV) using a Computrace 797 (Metrohm Nordic AB, Sweden) with a platinum rotating disc electrode (Pt-RDE), a platinum auxiliary electrode, and an Ag/AgCl/KCl reference electrode. Samples were added with no further processing at 100 μl to the reaction vial. The samples were homogenous and left unfiltered since the measured iron species are far smaller than 10 kD, and filtering would therefore not make a difference. The temperature was kept at 37 °C with a thermostat jacket, and a pH metre was connected to the cell (Metrohm Nordic AB, Sweden) for continuous monitoring of the PH. The electrolyte was NaClO_4_ (100 mM), and the peaks appeared at −0.145 and −0.461 A for Fe^3+^ and Fe^2+^, respectively. The pH of the electrolyte was kept at 4.0 to simulate the pH of the proximal part of the duodenum [19]. The method for running food samples (fermented carrot juices) was described previously [20].

### Statistics

Experimental values are presented as mean ± standard deviation (SD) or standard error of the mean (SEM) whenever applicable. Most food analyses were conducted in duplicates and are therefore not presented with ±SD. Cell experiments were conducted in triplicates or quadruplicates and repeated two times (three times in total). Values are presented as mean ± SD (*n* = 9 or *n* = 12). Statistical significance was calculated with Students’s two-tailed unpaired *t* test, and values <0.05 (*p* < 0.05) were considered as significant. Statistical analyses were performed in Microsoft Office Excel 2011 (for mac).

## Results

### Lactic-fermented vegetables added to a meal increased iron but not zinc absorption in humans

Iron absorption was significantly increased from 13.6 ± 1.6 to 23.6 ± 2.0 % (*p* < 0.0001) when fermented vegetables replaced fresh vegetables in the low-phytate meal and in the high-phytate meal from 5.2 ± 0.8 to 10.4 ± 2.4 % (*p* < 0.0001), Table 1. Zinc absorption from the low-phytate meal with added fresh or fermented vegetables was 42.3 ± 8.6 and 46.1 ± 10.0 %, respectively, and from the high-phytate meal 12.9 ± 3.5 and 13.4 ± 1.6 %, respectively. None of the changes in zinc absorption from meals with fresh or fermented vegetables were significant, Table 2.

**Table 1:** Iron absorption from meals with fresh and fermented vegetables

Meal	*n*	Non-heme iron content mg/serving (240 g)	Absorption %		Individual absorption^d^ A40 %		Mean of individual absorption ratio F:C^e^	Myo-inositol hexaphosphate μmol/serving (mg phytate-P)
			Meal	Ref. dose	Mean	SEM		
Low-phytate meal								
White wheat rolls^a,f^ + fresh vegetables^b^	8	4.4	9.5	25.0	13.6±	1.6	1.78 ± 0.10	6.1 (1.1)
White wheat rolls^a,f^ + fermented vegetables^b,f^	8	4.4	15.6	25.0	23.6±	2.0		2.5 (0.5)
High-phytate meal								
Wheat bran rolls^c,f^ + fresh vegetables^b,f^	9	4.0	4.6	36.8	5.2±	0.8	2.07 ± 0.16	159.2 (29.6)
Wheat bran rolls^c,f^ + fermented vegetables^b,f^	9	4.0	8.8	35.8	10.4±	2.4		155.6 (28.9)

^a^140 g white wheat rolls

^b^100 g fresh or fermented vegetables

^c^140 g wheat bran rolls

^d^Mean individual absorption ratios (test meal/reference dose) were multiplied by 40 to obtain the percentage absorption of iron corresponding to a 40 % reference dose absorption

^e^C = meal with fresh vegetables; F = meal with fermented vegetables

^f^All analytical measurements were taken in duplicates

**Table 2:** Zinc absorption from meals with fresh and fermented vegetables

Meal All meals incl. 50 g cottage cheese	*n*	Zinc content μmol/serving	Zinc absorption %		Absorption ratio F:C^d^	Myo-inositol hexaphosphate μmol/serving (mg phytate-P)
			Mean	SD		
Low-phytate meal						
White wheat rolls^a,e^ + fresh vegetables^b,e^	8	17.1	42.3 ±	8.6	1.09 ± 1.16	13.5 (2.5)
White wheat rolls^a,e ^+ fermented vegetables^b,e^	8	15.6	46.1 ±	10.0		6.3 (1.2)
High-phytate meal						
Wholemeal wheat rolls^c,e^ + fresh vegetables^b^	6	31.1	12.9 ±	3.5	1.04 ± 0.27	363.8 (67.6)
Wholemeal wheat rolls^c,e ^+ fermented vegetables^b^	6	32.4	13.4 ±	1.6		356.6 (66.3)

^a^100 g white wheat rolls

^b^200 g fresh or fermented vegetables

^c^100 g wholemeal wheat rolls

^d^C = meal with fresh vegetables; F = meal with fermented vegetables

^e^All analytical measurements were taken in duplicates

#### Composition of test meals

Iron, zinc, calcium, and phytate content in bread rolls and vegetables is presented in Table 3. Ascorbic acid (5.1 mg/g) and small amounts of phytate were present in the fresh vegetables but were undetectable in the fermented vegetable mix. The white bread rolls contained very low amounts of phytate (0.2 mg phytate-phosphorus/100 g). The lactic fermentation decreased the pH from 6 to 4 in the vegetable mix, and the major organic acids produced were lactic acid (10 mg/g), acetic acid (2 mg/g), and propionic acid (1.1 mg/g). The main organic acids in the fresh vegetables were malic acid (3.9 mg/g) and citric acid (0.7 mg/g). These acids were presumably consumed during the fermentation.

**Table 3:** Mineral and phytate content of the test meals (μmol/100 g)

Food sample	Ca mmol	Zn μmol	Fe μmol	Myo-inositol hexaphosphate μmol (mg phytate-P)
Fresh vegetables^a^	0.8	4.1	3.9	4.4 (0.8)
Fermented vegetables^b^	0.5	3.2	3.1	n. d.
Test meals iron absorption				
White wheat rolls	n. m.	8.4	68.0	1.2 (0.2)
Wheat bran rolls	n. m.	14.8	68.0	110.6 (20.5)
Test meals zinc absorption				
White wheat rolls	0.6	8.0	75.8	4.7 (0.9)
Wholemeal wheat rolls	0.8	24.0	38.0	355.0 (66.0)
Cottage cheese	1.0	5.4	1.8	–

All analytical measurements were made in duplicates

*n. d.* not detected

*n. m.* not measured

^a^Mean values of four different batches

^b^Mean values of two different batches

### Fermented vegetables provoked a greater hepcidin release in the Caco-2/HepG2 system

**Fig. 1:**
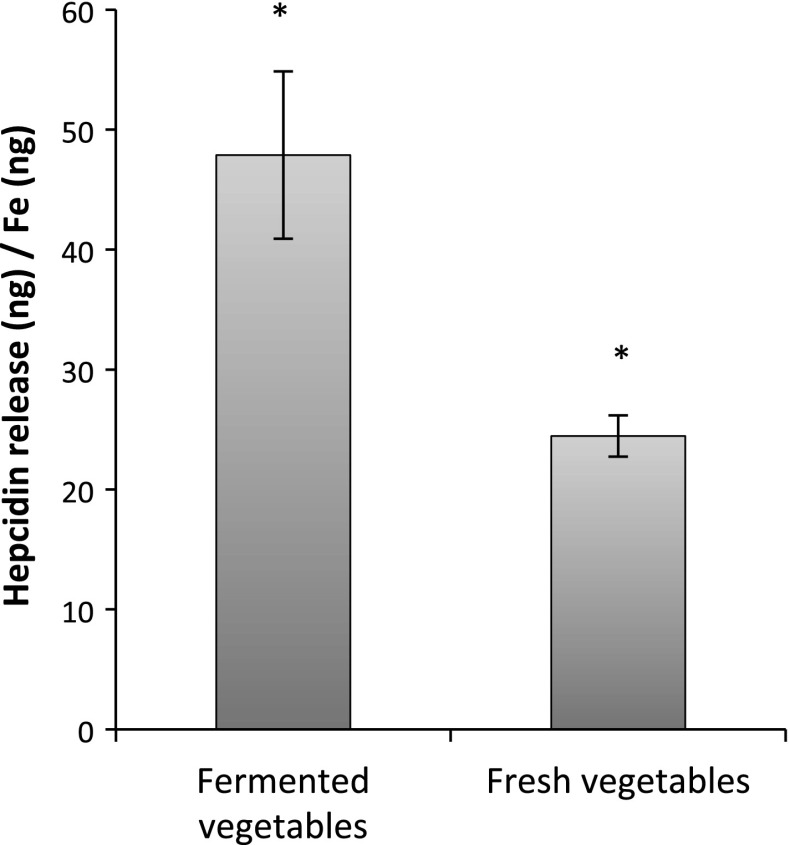
Hepcidin release from HepG2 cells of the Caco-2/HepG2 cell system, in response to fermented or fresh vegetables, normalized to soluble iron content of the respective treatment. Data are presented as mean ± SD (*n* = 9). An *asterisk* indicates a significant difference (*p* < 0.05)

In unfiltered in vitro digested vegetable mixes, the soluble iron content was higher in the fermented compared to the fresh mix (0.73 vs. 0.39 mg/L). However, after simulated mucus layer filtration (10 kD cut-off), the soluble iron content was higher in the fresh mix, suggesting that iron is more frequently associated with high molecular weight complexes (>10 kD) in the fermented mixes. This could imply that these complexes may not traverse the mucus layer. Despite about 40 % lower soluble iron content in the filtered vegetable mixes, the fermented vegetables were able to provoke a higher hepcidin response per ng of available iron (47.9 ± 6.9 ng/ng Fe vs. 24.5 ± 1.7 ng/ng Fe, *p* = 0.005, Fig. 1), indicating that Fe in the fermented mixes was more bioavailable compared to Fe in the fresh vegetables.

### Hydrated Fe^3+^ species increase after lactic fermentation and in vitro digestion

**Fig. 2:**
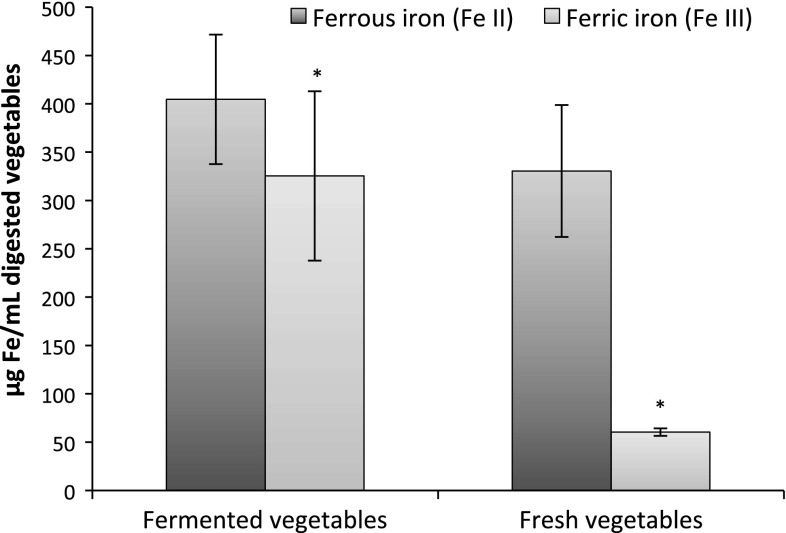
Differential pulse anodic stripping voltammetry measurements of Fe^2+^ and Fe^3+^ species in in vitro digested fermented and fresh vegetables. Data are presented as mean ± SD (*n* = 15). An *asterisk* indicates a significant difference (*p* < 0.05)

The reason for the increased hepcidin release could be that more iron reaches the basal compartment, and therefore, the hepcidin response was greater. In order to investigate this further, we measured the content of available ferrous (Fe^2+^) and ferric (Fe^3+^) iron in the digested unfiltered mixes by DP-ASV. The results revealed that available ferric iron [Fe(H_2_O)_6_]^3+^ was increased from 15 to 45 % of the total Fe^2+^ and Fe^3+^ pool (*p* = 0.04) in the digested fermented samples compared to the digested fresh samples, while the content of ferrous iron [Fe(H_2_O)_6_]^2+^ was unchanged (*p* = 0.63, Fig. 2). In previous studies on fermented carrot juice, we observed an increase in available ferric iron but, in contrast to the present study, did also observe a decrease in available ferrous iron in fermented carrot juice compared to fresh carrot juice [20]. However, the carrot juices were not exposed to simulated gastrointestinal digestion as the vegetables were in the present study.

### Lactate addition to Caco-2 cells did not increase intracellular ferritin

**Fig. 3:**
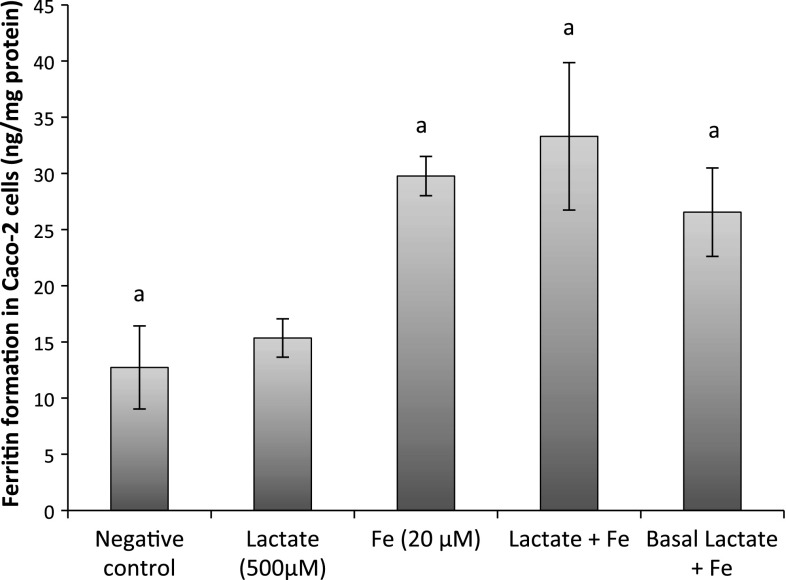
Ferritin levels in mono-cultured Caco-2 cells treated with lactate and/or Fe (20 μM). Data are presented as mean ± SD (*n* = 9). The *letter a* indicates a significant difference from negative control (*p* < 0.05)

In these experiments, the effect of lactate (Na lactate; 500 μM) on iron uptake (FeCl_2_·4H_2_O; 20 μM) was investigated in Caco-2 cells. Both the apical combination of lactate and iron and pre-incubation with lactate in the basal medium followed by iron incubation on the apical side were studied. The data showed that ferritin levels were increased in iron-treated cells by 133 % compared to untreated cells (*p* = 0.0002, Fig. 3). The addition of lactate did not significantly affect the ferritin formation in iron-treated cells independent of apical or basal addition. Nor did lactate without iron differ significantly from the baseline ferritin levels. The same experiments were done with lactate at 300 μM, showing the same trend (data not shown).

### Lactate did not affect hepcidin release

**Fig. 4:**
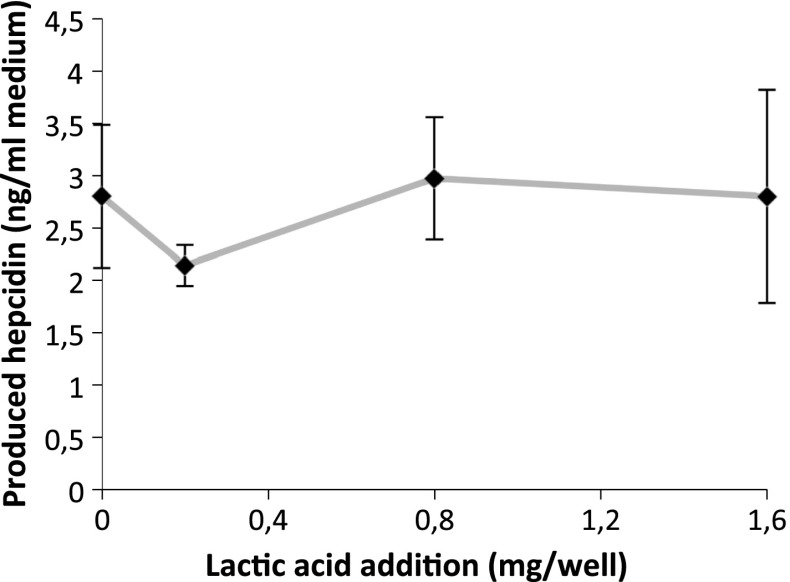
Hepcidin release from HepG2 cells in the Caco-2/HepG2 cell system in response to lactic acid (0–1.6 mg/well). The Caco-2 cells were treated with Fe (40 μM). Data are presented as mean ± SD (*n* = 12)

We also used the Caco-2/HepG2 cell model to investigate whether lactic acid could affect hepcidin production in liver cells even if ferritin formation in Caco-2 cells was unaffected by the organic acid. In the concentration range 0–1.6 mg lactic acid/well (0–32 mM) and Fe at 40 μM, no significant difference in hepcidin levels was observed compared to the baseline (at 0 mg lactate; *p* = 0.67, *p* = 0.76, and *p* = 0.8, respectively, Fig. 4).

## Discussion

### Improved iron absorption from lactic fermentation of vegetables

The phytate-phosphorus content was quite similar in the meals with wheat bran rolls with added fresh or fermented vegetables (29.6 vs. 28.9 mg), but the ascorbic acid content was higher (by 5 mg/g of total vegetables) in the fresh vegetable meal. Despite these circumstances, the fractional iron absorption was increased by approximately 100 % when the vegetables were fermented instead of fresh. This suggests that reduced phytate content of the vegetables did not play a major role in the promotion of iron absorption from fermented vegetables included in a phytate-containing meal. In addition, the fractional iron absorption from the wholemeal rolls with fermented vegetables (10.4 %) was much higher than could be expected from the phytate-phosphorus content, while the absorption from the whole-meal rolls with fresh vegetables (5.2 %) was as expected from a previously found relationship between iron absorption and phytate content [21, 22]. With a similar magnitude, fermented vegetables added to a white wheat roll meal (low phytate) improved iron absorption (approximately 75 %) compared to the addition of fresh vegetables.

### No effect of lactic fermentation of vegetables on zinc absorption

There are a few important aspects that may be the cause for not gaining an increase in zinc absorption after the lactic fermentation in the present study. First, zinc absorption is not regulated by means of redox states as in the absorption of iron; second, zinc is normally abundant in food in much lower concentrations than iron. This may imply that phytate, as an inhibitor of absorption, could be more important in zinc than iron absorption from fermented vegetables. In the present study, the meals in the zinc absorption study contained more than double the molar weight of phytate-phosphorus compared to the iron absorption study. In addition, the meals in the zinc absorption study included cottage cheese, which contributed with additional Ca^2+^ and protein. The combination of more phytate and Ca^2+^ could lead to calcium-phytate aggregates, which makes zinc less available for absorption. In addition, the additional protein may have an enhancing effect on zinc absorption, which could mask the effect of lactic fermentation on zinc absorption.

### Increased iron uptake despite decreased solubility

As we have shown previously, adding samples of filtered digested fermented or fresh carrot juices with an equal soluble iron content to mono-cultured Caco-2 cells does not cause significant differences in intracellular ferritin formation, an estimate of iron uptake. In the present study, the soluble iron content of filtered digested fermented vegetable mixes was *lower* than the iron in the fresh mixes, which implies that even if the total soluble iron content increases with the fermentation, it does not necessarily mean that more soluble iron reaches the cell surface of the absorptive enterocytes after the filtering of the mucus layer (10–15 kD cut-off). Therefore, solubility or availability may not be a correct estimate of iron uptake into intestinal cells. In the present study, we observed that lactic-fermented vegetables compared to fresh vegetables increased the ferritin formation and hepcidin release per each mole of Fe, despite that the filtered digested fermented vegetables contained less soluble iron than the fresh vegetables.

### The ferric to ferrous iron ratio is changed after fermentation

This may not come as a surprise since ferric iron is more stable in an acidic water solution than ferrous iron. Iron is absorbed in the gastrointestinal tract by a few different transport mechanisms regulated by several nutrients including trace metals [23]. For a few of the involved metals, including iron, the redox state is important for the regulation of absorption. Iron may be transported across the intestinal border as Fe^2+^ through the iron transporter DMT1, or heme-bound by HCP1 or, as lately proposed, by endocytosis of larger nano-particulate ferric iron complexes [24]. At least previously, there has been a common understanding that iron from vegetables is absorbed as ionic (elemental) iron and that Fe^2+^ is the preferred species. Ferric iron (Fe^3+^) may be reduced to Fe^2+^ by luminal enhancers of iron absorption such as ascorbic acid or by a membrane-bound extracellular reductase (Dcytb) located in the enterocyte membrane [25]. Although Fe^2+^ is the transported species, it may be favourable for iron absorption to have Fe^3+^ in the gastrointestinal passage since this species is less reactive than its reduced counterpart. In addition, the mucus layer binds Fe^3+^ under acidic conditions [26, 27], which may provide the reductase with its substrate. It has been shown that reductase activity is essential for iron absorption [28]. Another route for ferric iron absorption may be the spontaneous formation of polymeric ferric iron complexes in the gastrointestinal tract presumably absorbed by endocytosis. The increase in hydrated Fe^3+^ caused by the fermentation may therefore be beneficial for iron uptake.

### No effect of lactic acid on iron bioavailability

Lactic acid has been observed to have both positive and negative effects in addition to no effect at all on iron absorption in human studies [29]. It has been argued that lactic acid keeps iron soluble in the intestinal lumen by weakly associating with Fe. One could also argue that high levels of lactic acid in fermented products lower the pH, which is an enhancer of iron absorption in more than one aspect; the iron transporter DMT1 is driven by a pH gradient, in addition to that iron precipitation in hydroxy complexes is less favourable at lower pH values. However, when food iron reaches the intestinal surface, the pH is more or less neutral (6.8–7.4), which reduces the impact of pH on iron transcellular transport. In our studies, there was no pH difference between the digested fermented and fresh vegetables after in vitro digestion. The pH was also kept constant in the pure lactate/lactic acid experiments (7.1–7.4). We did not observe any effect of lactate on ferritin formation in the presence of iron (20 μM), independent of apical, basal, combined, or single dose addition, suggesting that lactate does not directly promote iron uptake by extracellular association. Neither did lactic acid at several doses in combination with the double dose of Fe (40 μM) affect hepcidin production in the Caco-2/HepG2 cell system.

## Conclusions

The increased bioavailability of iron from lactic-fermented vegetables added to a bread meal is unlikely to be an effect of the reduction of phytate, increased iron solubility, or the increase in lactic acid content. It is more likely an effect of the increase in hydrated ferric iron (Fe^3+^) species caused by the fermentation. Further studies on this theme are needed. In contrast, we did not observe any effects of lactic-fermented vegetables on zinc absorption, despite the inclusion of cottage cheese into the study and that the phytate level was higher in the fresh vegetables compared to the fermented ones.
